# Left Atrial Appendage Closure Versus Oral Anticoagulants in Atrial Fibrillation: A Systematic Review and Meta-Analysis

**DOI:** 10.3390/jcdd12120483

**Published:** 2025-12-08

**Authors:** Chen Wang, Dan Zhu, Jinliang Nan, Danyang Zhang

**Affiliations:** 1 Department of Cardiology, The Second Affiliated Hospital, School of Medicine, Zhejiang University, Hangzhou 310009, China; 11418143@zju.edu.cn (D.Z.); jinliangnan@zju.edu.cn (J.N.); danyangzhang@zju.edu.cn (D.Z.); 2 State Key Laboratory of Transvascular Implantation Devices, Hangzhou 310009, China; 3 Heart Regeneration and Repair Key Laboratory of Zhejiang Province, Hangzhou 310009, China

**Keywords:** atrial fibrillation, left atrial appendage closure, oral anticoagulants, systematic review, meta-analysis

## Abstract

Background: Left atrial appendage (LAA) closure is an alternative to oral anticoagulants (OAC) for stroke prevention in atrial fibrillation (AF), but comparative evidence remains inconsistent. This study systematically evaluates the efficacy and safety of LAA closure versus OAC in AF patients. Methods: We systematically searched PubMed, EmBase, Cochrane Library, and Web of Science for randomized controlled trials (RCTs) and propensity score-matched (PSM) studies published up to 30 September 2025. Treatment effects were estimated using relative risks (RR) with 95% confidence intervals (CI), and a random-effects model was applied for all analyses. Results: Fifteen studies (17,116 AF patients) were included, comprising 4 RCTs, 3 prospective PSM studies, and 8 retrospective PSM studies. Compared with OAC, LAA closure significantly reduced the composite endpoint (RR: 0.79; 95% CI: 0.66–0.95; *p* = 0.010), all-cause mortality (RR: 0.58; 95% CI: 0.49–0.69; *p* < 0.001), and cardiovascular mortality (RR: 0.55; 95% CI: 0.44–0.67; *p* < 0.001). Risks of any stroke (RR: 1.06; 95% CI: 0.86–1.31; *p* = 0.555), ischemic stroke (RR: 1.00; 95% CI: 0.85–1.17; *p* = 0.972), hemorrhagic stroke (RR: 0.96; 95% CI: 0.54–1.70; *p* = 0.879), and major bleeding (RR: 0.84; 95% CI: 0.67–1.04; *p* = 0.112) were not significantly different between groups. Conclusions: In AF patients, LAA closure significantly reduces mortality and a composite clinical endpoint compared to OAC, with similar risks of stroke and major bleeding. It is a favorable alternative for patients unsuitable for long-term anticoagulation.

## 1. Introduction

Atrial fibrillation (AF), the most common sustained cardiac arrhythmia worldwide, poses a growing epidemiological burden fueled by an aging population and the rising prevalence of chronic diseases [1]. According to the Global Burden of Disease Study, over 37 million individuals were affected by AF globally in 2017 [2]. The principal pathological risk of AF stems from the loss of effective atrial contraction, promoting blood stasis within the left atrial appendage (LAA) and establishing it as the predominant site for thrombus formation [3]. In non-valvular AF, approximately 90% of thrombi originate in the LAA [4]. AF is independently associated with an increased risk of heart failure, stroke, and all-cause mortality [5,6], and constitutes a major driver of escalating healthcare costs. Recent decades have witnessed an exponential rise in AF-related hospitalizations, imposing a substantial economic strain on healthcare systems worldwide [7,8].

For over half a century, oral anticoagulants (OACs) have represented the cornerstone for thromboembolism prevention in AF, with their efficacy robustly demonstrated in extensive clinical trials. Although traditional vitamin K antagonists (VKAs) significantly reduce stroke risk, their use necessitates regular international normalized ratio (INR) monitoring to maintain a therapeutic balance between efficacy and bleeding risk. In real-world practice, however, only about half of patients achieve adequate time-in-therapeutic-range (TTR) [9]. The introduction of non-vitamin K antagonist oral anticoagulants (NOACs) has mitigated some limitations of VKAs, yet the risk of bleeding complications persists. This risk is particularly pronounced in elderly patients (≥75 years) and those with comorbidities such as chronic kidney disease or peptic ulcer disease [10]. Furthermore, suboptimal long-term medication adherence remains a critical challenge, significantly influencing clinical outcomes [11].

Given these limitations of pharmacotherapy, LAA closure has emerged as an interventional strategy for the non-pharmacological “source control” of thrombi [12]. The success and safety of the LAAC procedure are highly dependent on meticulous pre-procedural planning. This involves comprehensive multimodality imaging (e.g., transesophageal echocardiography, cardiac computed tomography) to accurately assess LAA anatomy, dimensions, and thrombotic status, which is crucial for appropriate patient selection and optimal device choice [13]. A multidisciplinary, heart-team approach is therefore fundamental to maximizing procedural outcomes and long-term efficacy. Nevertheless, current evidence directly comparing the efficacy and safety of LAA closure versus OAC remains inconclusive. This study therefore aims to systematically evaluate and compare the efficacy and safety profiles of LAA closure and OAC in AF patients by integrating all available global randomized controlled trials (RCTs) and propensity score-matched (PSM) studies.

## 2. Materials and Methods

### 2.1. Data Sources, Search Strategy, and Selection Criteria

This systematic review and meta-analysis were conducted in accordance with the Preferred Reporting Items for Systematic Reviews and Meta-Analyses (PRISMA) guidelines [14]. The study protocol was registered on the INPLASY platform (Registration number: INPLASY2025100093).

A comprehensive literature search was performed in PubMed, Embase, the Cochrane Library, and Web of Science for studies published from inception until 30 September 2025. The search strategy utilized a combination of the following key terms: “Atrial Fibrillation,” “Left Atrial Appendage Closure,” “Oral Anticoagulants,” “Randomized Controlled Trial,” and “Propensity Score Matching.” The detailed search syntax for each database is provided in Supplementary Section S1. We also searched ClinicalTrials.gov for relevant studies with posted results that met our eligibility criteria. Additionally, the reference lists of all included articles were manually reviewed to identify any additional pertinent publications.

Two investigators independently screened all retrieved records. Any disagreements during the screening process were resolved through consensus or by consultation with a third reviewer when necessary. The inclusion criteria were: (1) study design: RCTs or PSM studies (prospective or retrospective); (2) population: patients diagnosed with AF requiring long-term thromboprophylaxis; (3) intervention: LAA closure (intervention group) versus OAC therapy (control group, including VKAs or NOACs); and (4) minimum follow-up duration of ≥3 months and reporting of at least one relevant clinical outcome (composite endpoint, any stroke, ischemic stroke, hemorrhagic stroke, all-cause mortality, cardiac death, or major bleeding). Exclusion criteria comprised: (1) non-comparative studies; (2) study populations with acute AF, post-operative AF, or active thrombosis/bleeding; (3) interventions involving non-percutaneous LAA closure or control groups receiving antiplatelet monotherapy; and (4) studies with incomplete or unavailable outcome data.

### 2.2. Data Collection and Quality Assessment

A pre-piloted, standardized data extraction form was used by two independent investigators to collect the following data from each included study: first author, publication year, study design, sample size, patient baseline characteristics (mean age, male proportion, comorbidities including diabetes mellitus, hypertension, coronary artery disease, congestive heart failure, prior stroke), CHA_2_DS_2_-VASc score, details of the intervention and control regimens, and composite endpoint definition, follow-up duration, and all reported outcomes. The extracted data were cross-checked to ensure accuracy. The Cochrane Risk of Bias tool was employed to assess the methodological quality of the included RCTs [15], while the Newcastle-Ottawa Scale (NOS) was used for the PSM studies [16]. Any discrepancies in data extraction or quality assessment were resolved through discussion or by adjudication from a third investigator.

### 2.3. Statistical Analysis

Relative risk (RR) with 95% confidence interval (CI) was used as the summary measure for all dichotomous outcomes. A random-effects model was applied for all meta-analyses to account for potential clinical and methodological heterogeneity across studies [17,18]. Heterogeneity was quantitatively assessed using the *I*^2^ statistic and Cochran’s Q test, with *I*^2^ > 50% or a *p*-value for the Q test < 0.10 indicating substantial heterogeneity [19,20]. Sensitivity analyses were performed to evaluate the robustness of the pooled results [21]. Pre-specified subgroup analyses were conducted for the composite endpoint and major bleeding based on the following variables: study design, mean age, proportion of males, prevalence of diabetes, hypertension, coronary artery disease, congestive heart failure, prior stroke, CHA_2_DS_2_-VASc score, type of OAC control, and follow-up duration. The differences between subgroups were examined using interaction tests [22]. To further explore significant heterogeneity, univariate random-effects meta-regression analyses were pre-planned [23]. Potential publication bias was assessed visually using funnel plots and statistically using Egger’s and Begg’s tests for investigated outcomes [24,25]. If significant publication bias was detected, the trim-and-fill method was applied to adjust the effect estimate [26]. A two-sided *p*-value < 0.05 was considered statistically significant for all tests, except where otherwise specified for heterogeneity. All statistical analyses were performed using Stata version 18.0 (StataCorp LLC, College Station, TX, USA).

## 3. Results

### 3.1. Literature Search

**Figure 1:**
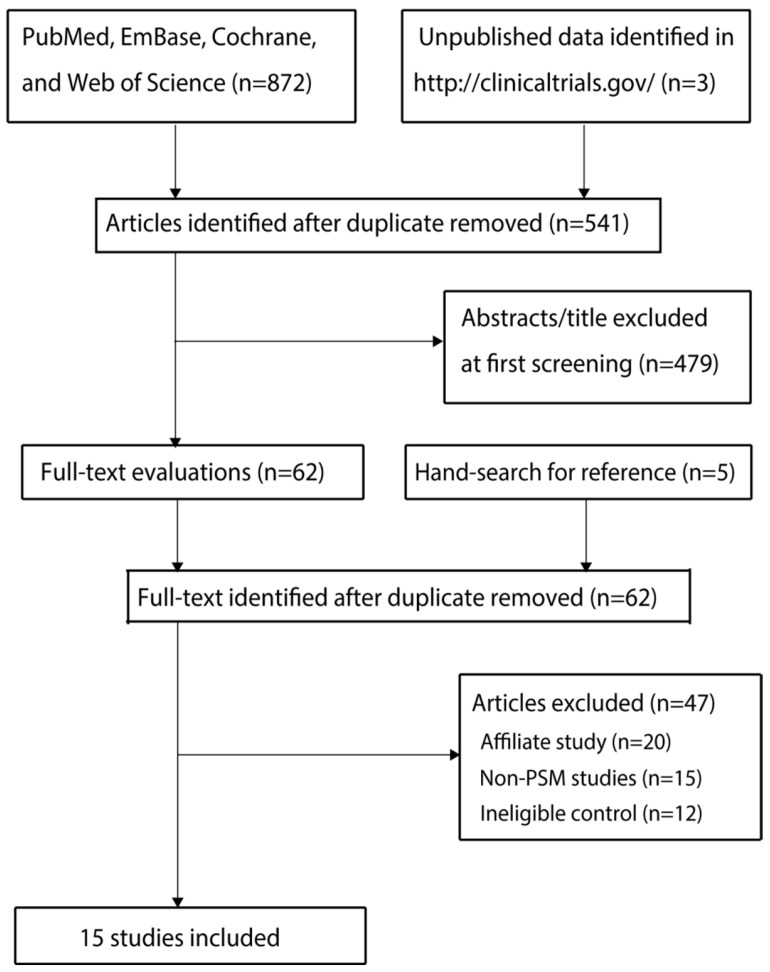
Study Selection Flowchart. A flow diagram illustrating the process of study identification, screening, eligibility assessment, and final inclusion, in accordance with the Preferred Reporting Items for Systematic Reviews and Meta-Analyses (PRISMA) statement.

The initial electronic database search identified 872 records. After the removal of 331 duplicates, 541 unique records were screened based on their titles and abstracts. Of these, 479 publications were excluded for being irrelevant to the research topic or not matching the eligible study types, leaving 62 articles for full-text review. Upon detailed assessment, 47 studies were excluded for the following reasons: duplicate or overlapping patient cohorts (n = 20), non-PSM study design (n = 15), and ineligible control groups (n = 12). Consequently, 15 studies [27,28,29,30,31,32,33,34,35,36,37,38,39,40,41] fulfilling all pre-specified eligibility criteria were included in the final meta-analysis. A manual search of the reference lists of these included studies did not yield any additional eligible publications. The study selection process, detailed in Figure 1, was conducted in strict adherence to the PRISMA guidelines.

### 3.2. Study Characteristics and Quality Assessment

The baseline characteristics of the 15 included studies are summarized in Table 1. The final analysis comprised 4 RCTs, 3 prospective PSM studies, and 8 retrospective PSM studies, encompassing a total of 17,116 patients with AF. The sample sizes of individual studies ranged from 116 to 4410, with follow-up durations spanning from 1 to 3.8 years. The mean age of participants across the studies ranged from 69.5 to 85.7 years, and males constituted 52.7% to 70.3% of the populations. The mean CHA_2_DS_2_-VASc scores varied from 3.5 to 5.7. Regarding the control intervention, two studies used VKAs, ten used NOACs, and the remaining three studies used OACs as the control group.

The results of the methodological quality assessment are detailed in Supplementary Tables S1 and S2. Among the four RCTs, all were judged to be at low risk of bias, except for the PROTECT AF trial, which raised some concerns regarding blinding in outcome assessment. For the eleven included PSM studies, eight received a high-quality score of 9 stars on the NOS, and three were awarded 8 stars.

### 3.3. Composite Endpoint

**Figure 2:**
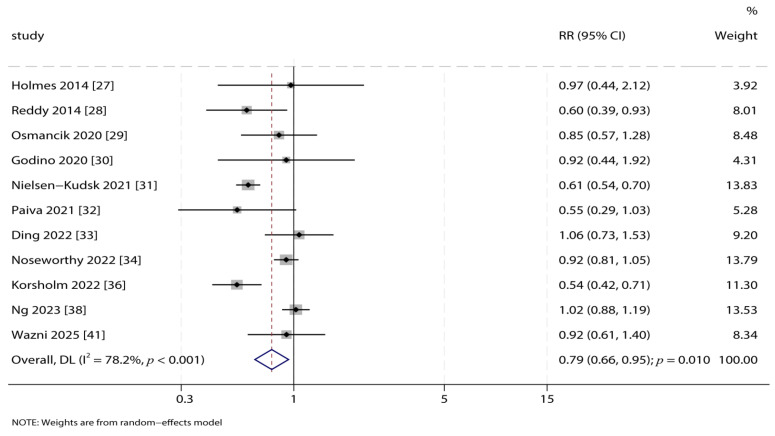
Forest Plot for the Composite Endpoint. Forest plot comparing the risk of the composite endpoint (ischemic stroke + systemic embolism + all-cause mortality) between left atrial appendage (LAA) closure and oral anticoagulant (OAC) groups. The pooled relative risk (RR) with 95% confidence interval (CI) was calculated using a random-effects model [27,28,29,30,31,32,33,34,36,38,41].

Pooled data from eleven studies reporting the composite endpoint demonstrated that LAA closure was associated with a statistically significant 21% reduction in risk compared to OAC therapy (RR: 0.79; 95% CI: 0.66–0.95; *p* = 0.010; Figure 2). Significant heterogeneity was observed among these studies (*I*^2^ = 78.2%, *p* < 0.001). Sensitivity analysis confirmed that this pooled result was robust and not substantially influenced by the sequential exclusion of any single study (Supplementary Figure S1). Pre-specified subgroup analyses revealed that the benefit of LAA closure in reducing the composite endpoint was particularly evident in several subgroups, including prospective PSM studies, patients with a mean age ≥ 75.0 years, and those with a mean CHA_2_DS_2_-VASc score ≥ 4.0. Statistically significant interactions, indicating differential treatment effects across subgroups, were observed for study design and the proportion of patients with diabetes (Table 2). To quantitatively explore the substantial heterogeneity observed for the composite endpoint, chronic heart failure proportion (*p* = 0.095) might have played contributed an important role in LAA closures vs. OAC on the risk of composite endpoint (Supplementary Figures S2–S11).

### 3.4. Any Stroke, Ischemic Stroke, and Hemorrhagic Stroke

**Figure 3:**
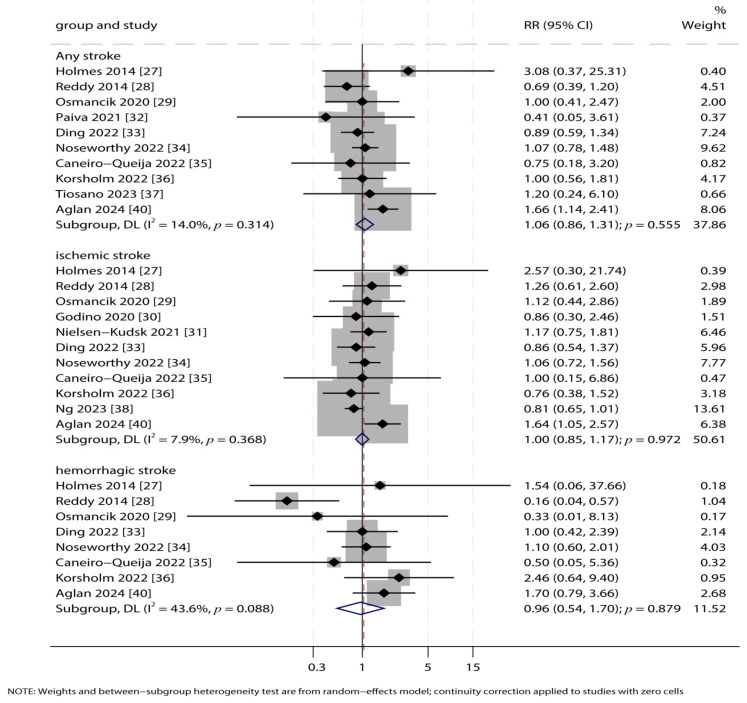
Forest Plots for Stroke Outcomes. Forest plots comparing the risks of any stroke, ischemic stroke, and hemorrhagic stroke between LAA closure and OAC groups. Pooled relative risks (RR) with 95% confidence intervals (CI) were calculated using a random-effects model [27,28,29,30,31,32,33,34,35,36,37,38,40].

Data on any stroke, ischemic stroke, and hemorrhagic stroke were reported in 11, 10, and 8 studies, respectively. Meta-analysis showed no significant differences in the risks of any stroke (RR: 1.06; 95% CI: 0.86–1.31; *p* = 0.555), ischemic stroke (RR: 1.00; 95% CI: 0.85–1.17; *p* = 0.972), or hemorrhagic stroke (RR: 0.96; 95% CI: 0.54–1.70; *p* = 0.879) between the LAA closure and OAC groups (Figure 3). Heterogeneity was low for any stroke (*I*^2^ = 14.0%, *p* = 0.314) and ischemic stroke (*I*^2^ = 7.9%, *p* = 0.368), while moderate heterogeneity was noted for hemorrhagic stroke (*I*^2^ = 43.6%, *p* = 0.088).

### 3.5. All-Cause Mortality and Cardiac Death

**Figure 4:**
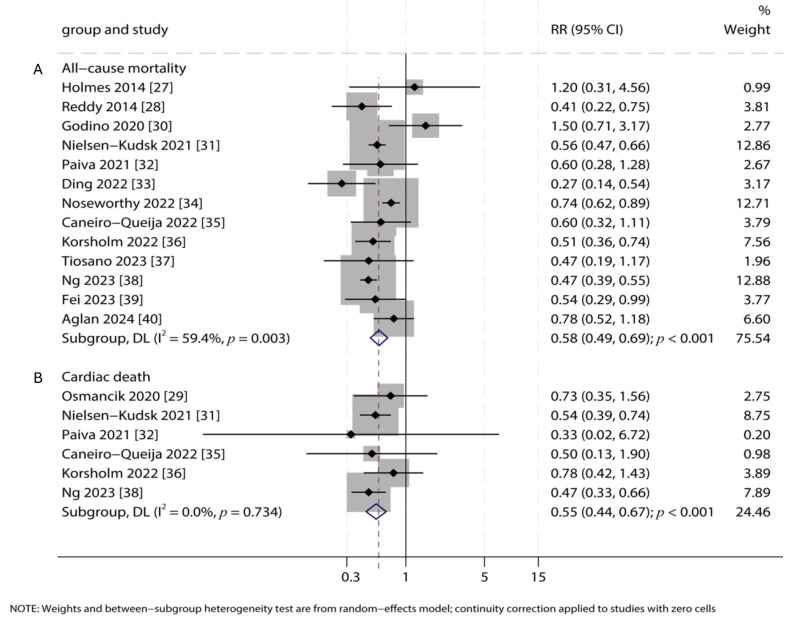
Forest Plots for Mortality Outcomes. Forest plots comparing the risks of (**A**) all-cause mortality and (**B**) cardiac mortality between LAA closure and OAC groups. Pooled relative risks (RR) with 95% confidence intervals (CI) were calculated using a random-effects model [27,28,29,30,31,32,33,34,35,36,37,38,39,40].

Thirteen studies reported data on all-cause mortality and six on cardiac death. The analysis revealed that LAA closure significantly reduced the risk of all-cause mortality by 42% (RR: 0.58; 95% CI: 0.49–0.69; *p* < 0.001) and cardiac death by 45% (RR: 0.55; 95% CI: 0.44–0.67; *p* < 0.001) compared to OAC (Figure 4). Significant heterogeneity was present for all-cause mortality (*I*^2^ = 59.4%, *p* = 0.003), but not for cardiac death (*I*^2^ = 0.0%, *p* = 0.734).

### 3.6. Major Bleeding

**Figure 5:**
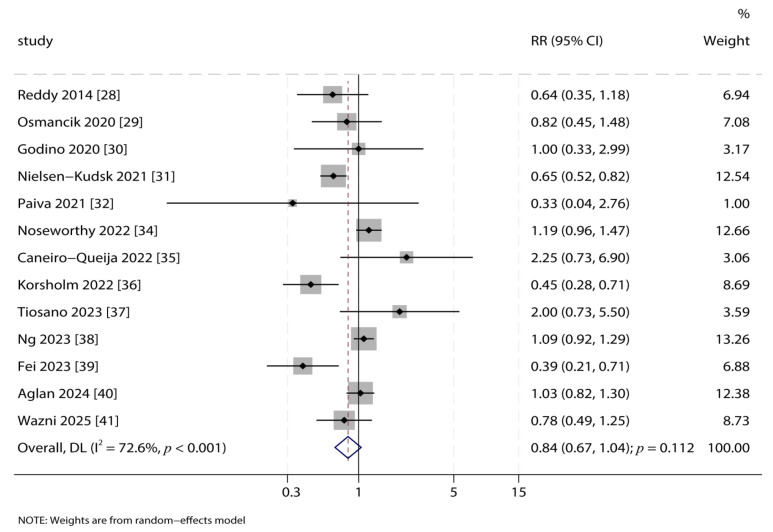
Forest Plot for Major Bleeding. Forest plot comparing the risk of major bleeding between LAA closure and OAC groups. The pooled relative risk (RR) with 95% confidence interval (CI) was calculated using a random-effects model [28,29,30,31,32,34,35,36,37,38,39,40,41].

Thirteen studies reported on major bleeding. The analysis indicated a trend towards a lower risk of major bleeding with LAA closure compared to OAC, although the result was not statistically significant (RR: 0.84; 95% CI: 0.67–1.04; *p* = 0.112; Figure 5). The included studies exhibited significant heterogeneity (*I*^2^ = 72.6%, *p* < 0.001). The sensitivity analysis confirmed the robustness of this finding (Supplementary Figure S12). Subgroup analyses suggested that LAA closure significantly reduced the risk of major bleeding in specific patient subsets, such as those enrolled in prospective PSM studies and those with a follow-up duration of ≥2.0 years. A significant subgroup interaction was observed for the proportion of patients with diabetes (Table 2). To quantitatively explore the substantial heterogeneity observed for the major bleeding, we noted mean age (*p* = 0.086), diabetes mellitus proportion (*p* = 0.058), hypertension proportion (*p* = 0.058), chronic heart failure proportion (*p* = 0.075), and stroke proportion (*p* = 0.075) might have played an important role in LAA closure vs. OAC on the risk of major bleeding (Supplementary Figures S13–S22).

### 3.7. Publication Bias

**Figure 6:**
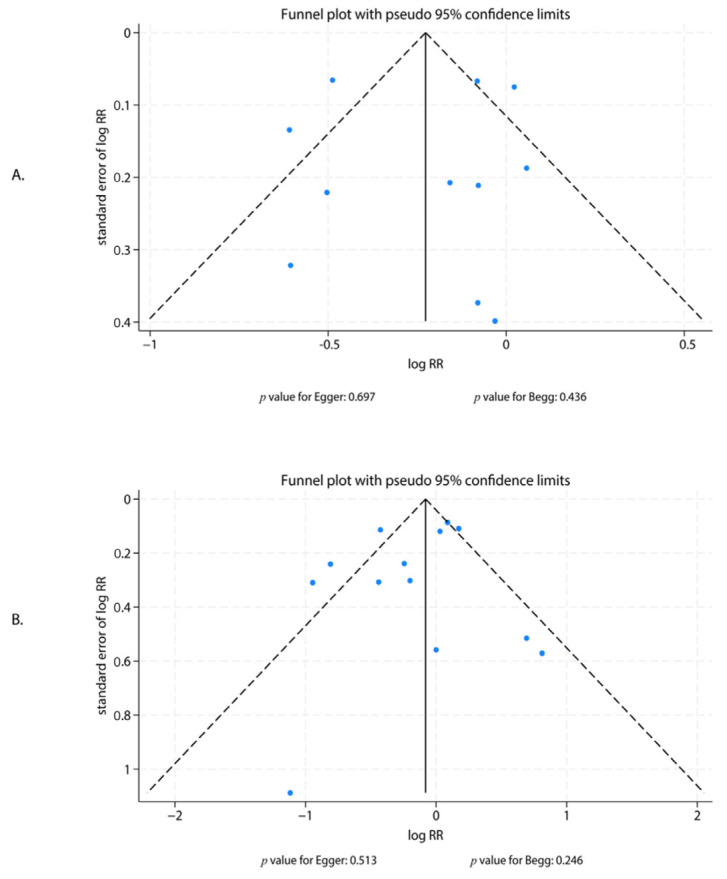
Funnel Plots for Publication Bias Assessment. Funnel plots evaluating potential publication bias for (**A**) the composite endpoint and (**B**) major bleeding. Statistical symmetry was assessed using Egger’s and Begg’s tests.

Visual inspection of the funnel plots and statistical evaluation using Egger’s and Begg’s tests did not reveal significant evidence of publication bias for either the composite endpoint (Egger’s test *p* = 0.697; Begg’s test *p* = 0.436) or major bleeding (Egger’s test *p* = 0.513; Begg’s test *p* = 0.246) (Figure 6).

## 4. Discussion

This large-scale, high-quality systematic review and meta-analysis, which integrated 15 studies including 4 low-risk-of-bias RCTs and 11 high-quality PSM studies involving 17,116 patients with AF, provides a comprehensive comparison of LAA closure and OAC. The principal finding is that LAA closure is significantly superior to OAC in reducing the composite endpoint, all-cause mortality, and cardiac mortality, while demonstrating comparable effects for stroke prevention and major bleeding. Furthermore, subgroup analyses identified specific patient profiles that derive the greatest benefit from LAA closure, offering crucial evidence for personalizing AF management strategies.

This study found that LAA closure reduced the risk of all-cause mortality by 42% and cardiac mortality by 45%. However, these findings should be interpreted with caution, and the association should not be construed as definitive proof of causality. The similar rates of stroke and major bleeding between groups suggest that the mortality benefit may not be solely driven by a reduction in these classic, direct complications of AF. The benefit is likely multifactorial. Mechanistically, LAA closure physically prevents thrombus formation, thereby circumventing the risk of fatal bleeding (e.g., intracranial hemorrhage) or devastating thromboembolic events, even if the overall rates of these events were not significantly different. The significant reduction in cardiac mortality may also be linked to indirect cardioprotective effects, such as improved hemodynamics and reduced neurohormonal activation from the excluded LAA [42]. Critically, the observed mortality benefit may be influenced by several unmeasured biases inherent in the included studies, particularly the non-randomized PSM cohorts. These include ‘healthier-user bias’, where patients selected for an invasive procedure like LAA closure may be systematically healthier or have better overall care access than those managed with pharmacotherapy alone. ‘Procedural selection bias’ is also a key factor, as clinicians meticulously select patients for LAA closure who are deemed to have a favorable anatomy and a high probability of surviving the procedure, potentially creating a cohort with a better inherent prognosis. Furthermore, ‘survivor bias’ is introduced because patients must be stable enough to undergo the procedure, automatically excluding those with imminent mortality. While propensity score matching adjusts for known confounders, it cannot fully account for these subtle selection biases, which may partly explain the large effect sizes observed for mortality.

The significant benefit in the composite endpoint underscores the comprehensive clinical value of the procedure. Subgroup analyses revealed that this benefit was most pronounced in prospective PSM studies, populations with a diabetes prevalence <30%, and those with a hypertension prevalence ≥85%. In diabetic patients, systemic endothelial dysfunction and a higher burden of non-LAA thrombogenic sources may diminish the relative advantage of a localized intervention like LAA closure [43]. Conversely, hypertensive patients often exhibit more significantly enlarged and dysfunctional LAAs with a higher inherent thrombotic risk, thus experiencing greater absolute risk reduction from closure [43]. These findings provide concrete evidence for refining patient selection.

Notably, our analysis found no statistically significant differences between LAA closure and OAC in the risks of any stroke, ischemic stroke, hemorrhagic stroke, or major bleeding, a finding consistent with previous reports [44]. This equivalence in stroke prevention, despite the LAA being the dominant source of thrombi (accounting for ~90% in non-valvular AF), can be explained by the fundamental difference in the mechanism of action [4]. OAC provides systemic anticoagulation, mitigating thrombotic risk from all potential sources, whereas LAA closure is a targeted mechanical intervention. Although the reduction in major bleeding with LAA closure was not statistically significant in the overall population, the significant risk reduction observed in subgroups like those with longer follow-up (≥2 years) is clinically critical. Bleeding risk with OAC is cumulative over time, while LAA closure typically necessitates only short-term post-procedural anticoagulation, leading to a sustained reduction in long-term bleeding risk [45]. This “time-dependent” benefit suggests that the advantage of LAA closure in reducing major bleeding becomes more apparent with extended observation.

The post-procedural antithrombotic regimen is a critical factor influencing the outcomes of LAA closure. In the included studies, patients typically received a short course of dual antiplatelet therapy [27,28,29] followed by long-term single antiplatelet therapy [37,39]. This strategy fundamentally differentiates LAA closure from lifelong OAC and is a likely contributor to the observed long-term reduction in major bleeding, as it limits the period of highest bleeding risk. However, this very strategy also introduces specific risks. In the initial period after device implantation, before endothelialization is complete, patients are at an increased risk of device-related thrombus (DRT), which can lead to ischemic stroke. The comparable rates of ischemic stroke between the LAA closure and OAC groups in our analysis suggest that the benefit of eliminating the LAA as a thrombogenic source is balanced by this early, transient risk of DRT. Variability in antithrombotic protocols across studies—in terms of drug choice (e.g., aspirin-clopidogrel vs. direct OAC), intensity, and duration—could be a significant source of the heterogeneity observed in our results for both bleeding and ischemic outcomes. Future research aimed at standardizing and personalizing the post-LAA closure antithrombotic strategy is essential to maximize the procedure’s safety and efficacy profile.

Significant heterogeneity was observed for several outcomes, particularly the composite endpoint and major bleeding. Our subgroup analyses indicated that study design and diabetes prevalence were major contributors. RCTs typically enroll more homogeneous patient populations under controlled conditions, whereas PSM studies often reflect the complexity of real-world practice, leading to variations in effect estimates. Furthermore, the heterogeneity in control interventions—with studies using either VKAs or the newer NOACs—likely played a role. Since NOACs are associated with a lower inherent bleeding risk than VKAs [10], their use as a comparator may attenuate the apparent safety advantage of LAA closure. Importantly, sensitivity analyses confirmed the stability of the pooled estimates, and no significant publication bias was detected, supporting the robustness of our primary conclusions.

The technical success of the LAA closure procedure itself is paramount to achieving good long-term outcomes. Recent evidence underscores that the implantation technique directly impacts procedural efficacy. As highlighted by the study from La Fazia et al. [46], device compression is a critical modifiable factor. Their findings demonstrated that a more compressed device implant (overcompression) was independently associated with a significantly lower rate of residual peri-device leak (PDL) at follow-up, without an increase in procedural complications. This is crucial because residual PDL has been linked to a higher risk of subsequent thromboembolic events. This evidence reinforces that the mortality benefits observed in our meta-analysis are contingent upon a high-quality, optimized implantation procedure that ensures complete LAA sealing. It also suggests that the results from high-volume centers with rigorous implant technique standards, as included in our analysis, may not be universally generalizable, and continuous refinement of implantation protocols is essential to maximize patient prognosis.

Notwithstanding the rigorous methodology adhering to PRISMA guidelines, this study has several limitations. First, although subgroup analyses explained some of the observed heterogeneity, residual heterogeneity likely persists due to unmeasured confounders, such as variations in outcome definitions and differences in the type of LAA closure device and operator experience. Second, the follow-up duration of the included studies was relatively short (1–3.8 years), with only four studies reporting outcomes beyond 3 years. This limits the assessment of the very long-term safety, device durability, and sustained survival benefits of LAA closure. Third, significant variability was observed in the definitions of the composite endpoint across studies. We acknowledge that a sensitivity analysis restricted to studies with a uniform definition would have been methodologically ideal. However, such an analysis was not feasible as the largest subgroup sharing an identical endpoint comprised only three studies, which would have provided an underpowered and unreliable estimate. Finally, as a study-level meta-analysis, the lack of individual patient data precluded more granular, patient-level risk stratification to identify which individuals are most likely to benefit.

## 5. Conclusions

This large-sample, high-quality meta-analysis suggests that in patients with AF, LAA closure is associated with a significant reduction in the composite endpoint, all-cause mortality, and cardiovascular mortality compared to oral anticoagulation, while maintaining comparable efficacy for stroke prevention and a trend towards reduced major bleeding. Subgroup analyses further indicate that older patients, those with high thrombotic burden, and populations with a lower prevalence of diabetes derive greater absolute benefit. These findings provide robust evidence supporting LAA closure as a valuable therapeutic alternative, particularly in the contemporary era of widespread NOAC use, offering a crucial option for high-risk populations. Future research should focus on long-term follow-up and individualized data analysis to further refine patient selection and advance AF stroke prevention towards a more precise and personalized paradigm.

## Figures and Tables

**Table 1:** The baseline characteristics of included studies and involved patients.

Study	Study Design	Sample Size	Age (Years)	Male (%)	DM (%)	HTN (%)	CAD (%)	CHF (%)	Stroke (%)	CHA DS_2_- VASc Score	Disease Status	Intervention	Control	Composite Endpoint Definition	Follow-Up (yrs)
Holmes, 2014 [27]	RCT	407	74.3	70.0	32.4	91.4	NA	23.3	27.8	3.8	Nonvalvular AF	LAA closure with Watchman (81–325 mg aspirin and 75 mg clopidogrel)	Warfarin (INR 2–3)	All-cause mortality, stroke, and SE	1.5
Reddy, 2014 [28]	RCT	707	72.0	70.3	26.2	89.8	NA	26.9	18.5	3.5	Nonvalvular AF	LAA closure with Watchman (81–325 mg aspirin and 75 mg clopidogrel)	Warfarin (INR 2–3)	All-cause mortality, stroke, and SE	3.8
Osmancik, 2020 [29]	RCT	402	73.3	65.7	40.5	92.5	17.2	44.3	32.7	4.7	Nonvalvular AF	LAA closure with Amulet, Watchman, or Watchman FLX (100 mg aspirin and 75 mg clopidogrel)	NOAC (apixaban, dabigatran, rivaroxaban)	Stroke, TIA, SE, major bleeding, cardiovascular death, significant complications	1.7
Godino, 2020 [30]	Pro PSM	192	74.6	68.8	24.5	88.5	12.6	NA	32.3	4.3	Nonvalvular AF	LAA closure with Amulet, Watchman	NOAC (apixaban, dabigatran, rivaroxaban)	IS, TIA, SE, AMI	2.0
Nielsen-Kudsk, 2021 [31]	Pro PSM	2255	75.1	62.7	33.6	85.1	33.2	17.8	31.4	4.3	Nonvalvular AF	LAA closure with Amulet	NOAC (not assigned)	IS, major bleeding, and all-cause mortality	2.0
Paiva, 2021 [32]	Pro PSM	240	76.6	53.3	28.3	84.2	14.6	31.7	32.1	4.9	Nonvalvular AF	LAA closure	NOAC (not assigned)	All-cause mortality, stroke, and major bleeding	1.1
Ding, 2022 [33]	Retro PSM	1322	69.6	66.0	32.7	69.4	57.4	36.2	6.9	NA	Nonvalvular AF	LAA closure	NOAC (apixaban, dabigatran, edoxaban, rivaroxaban)	Stroke, VT, and all-cause mortality	2.0
Noseworthy, 2022 [34]	Retro PSM	4410	75.8	54.8	52.5	98.2	76.3	56.3	33.6	5.7	Nonvalvular AF	LAA closure	NOAC (apixaban, dabigatran, edoxaban, rivaroxaban)	IS, SE, major bleeding, and all-cause mortality	1.5
Caneiro-Queija, 2022 [35]	Retro PSM	116	85.7	54.3	31.0	84.5	19.0	36.2	25.9	4.7	Nonvalvular AF	LAA closure	NOAC (not assigned)	Not reported	2.0
Korsholm, 2022 [36]	Retro PSM	587	76.2	66.6	28.6	84.0	27.4	13.8	28.8	5.3	Nonvalvular AF	LAA closure	NOAC (not assigned)	IS, major bleeding and all-cause mortality	2.0
Tiosano, 2023 [37]	Retro PSM	456	77.3	59.6	41.0	82.7	51.5	36.8	38.6	4.0	Nonvalvular AF	LAA closure with Amulet, Watchman (aspirin and clopidogrel for 6 weeks, then aspirin monotherapy)	NOAC (apixaban, dabigatran, rivaroxaban)	Not reported	1.0
Ng, 2023 [38]	Retro PSM	2350	75.9	57.4	30.2	67.7	45.1	29.1	39.2	4.5	Nonvalvular AF	LAA closure	NOAC (apixaban, dabigatran, edoxaban, rivaroxaban)	IS, major bleeding and all-cause mortality	2.9
Fei, 2023 [39]	Retro PSM	1364	70.3	52.7	21.8	70.3	40.6	28.0	25.3	3.5	Nonvalvular AF	LAA closure with Watchman (aspirin and clopidogrel for 3 months, then aspirin monotherapy)	OAC (warfarin, apixaban, dabigatran, edoxaban, rivaroxaban)	Not reported	3.1
Aglan, 2024 [40]	Retro PSM	708	72.7	64.5	42.8	89.4	24.7	NA	37.7	NA	Hypertrophic cardiomyopathy AF	LAA closure	OAC (warfarin, apixaban, dabigatran, edoxaban, rivaroxaban)	Not reported	3.0
Wazni, 2025 [41]	RCT	1600	69.5	65.8	NA	NA	NA	NA	NA	3.5	Nonvalvular AF	LAA closure	OAC (warfarin, apixaban, dabigatran, edoxaban, rivaroxaban)	All-cause mortality, stroke, and SE	3.0

Abbreviations: AF: atrial fibrillation; AMI: acute myocardial infarction; CAD: coronary artery disease; CHF: chronic heart failure; DM: diabetes mellitus; HTN: hypertension; IS: ischemic stroke; LAA: left atrial appendage; NA: not available; NOAC: non-vitamin K antagonist oral anticoagulants; OAC: oral anticoagulants; PSM: propensity score-matched; RCT: randomized controlled trial; SE: systemic embolism; TIA: transient ischemic attack; VT: venous thromboembolism.

**Table 2:** Subgroup analyses for composite endpoint and major bleeding.

Outcomes	Factors	Subgroups	RR and 95%CI	*p* Value	*I^2^* (%)	Q Statistic	Interaction Test
Composite endpoints	Study design	RCT	0.80 (0.64–1.01)	0.061	0.0	0.492	0.020
		Prospective PSM	0.62 (0.55–0.70)	<0.001	0.0	0.518	
		Retrospective PSM	0.86 (0.68–1.10)	0.231	83.1	<0.001	
	Mean age (yrs)	≥75.0	0.73 (0.56–0.95)	0.021	90.2	<0.001	0.295
		<75.0	0.87 (0.72–1.05)	0.152	0.0	0.549	
	Male (%)	≥65.0	0.78 (0.62–0.99)	0.041	50.5	0.059	0.969
		<65.0	0.79 (0.59–1.05)	0.107	90.8	<0.001	
	DM (%)	≥30.0	0.87 (0.70–1.10)	0.245	84.9	<0.001	0.014
		<30.0	0.58 (0.47–0.71)	<0.001	0.0	0.605	
	Hypertension (%)	≥85.0	0.77 (0.60–0.98)	0.032	76.0	<0.001	0.741
		<85.0	0.78 (0.53–1.14)	0.200	85.0	<0.001	
	CAD (%)	≥30.0	0.87 (0.66–1.14)	0.319	90.9	<0.001	0.373
		<30.0	0.66 (0.50–0.87)	0.004	34.5	0.205	
	CHF (%)	≥30.0	0.91 (0.79–1.05)	0.181	9.4	0.346	0.343
		<30.0	0.71 (0.52–0.97)	0.029	87.9	<0.001	
	Stroke history (%)	≥30.0	0.81 (0.64–1.03)	0.083	84.8	<0.001	0.706
		<30.0	0.73 (0.51–1.05)	0.094	68.1	0.024	
	CHA DS_2_- VASc score	≥4.0	0.76 (0.61–0.95)	0.018	85.5	<0.001	0.302
		<4.0	0.78 (0.58–1.06)	0.111	12.2	0.320	
	Control	VKA	0.68 (0.46–1.02)	0.061	6.7	0.301	0.583
		NOAC	0.79 (0.64–0.98)	0.029	83.9	<0.001	
		OAC	0.92 (0.61–1.40)	0.712	-	-	
	Follow-up (yrs)	≥2.0	0.77 (0.60–1.00)	0.046	84.1	<0.001	0.292
		<2.0	0.90 (0.80–1.02)	0.086	0.0	0.449	
Major bleeding	Study design	RCT	0.75 (0.55–1.03)	0.075	0.0	0.835	0.193
		Prospective PSM	0.66 (0.53–0.82)	<0.001	0.0	0.613	
		Retrospective PSM	0.92 (0.69–1.23)	0.587	78.3	<0.001	
	Mean age (yrs)	≥75.0	0.92 (0.65–1.28)	0.609	81.8	<0.001	0.425
		<75.0	0.76 (0.56–1.03)	0.076	49.8	0.076	
	Male (%)	≥65.0	0.66 (0.51–0.86)	0.002	6.0	0.373	0.066
		<65.0	0.94 (0.72–1.23)	0.632	77.8	<0.001	
	DM (%)	≥30.0	1.01 (0.81–1.28)	0.903	72.6	0.001	0.001
		<30.0	0.50 (0.37–0.67)	<0.001	0.0	0.516	
	Hypertension (%)	≥85.0	0.88 (0.67–1.15)	0.347	70.6	0.004	0.907
		<85.0	0.82 (0.46–1.46)	0.498	80.8	<0.001	
	CAD (%)	≥30.0	0.88 (0.62–1.25)	0.484	85.7	<0.001	0.755
		<30.0	0.83 (0.54–1.29)	0.416	62.7	0.020	
	CHF (%)	≥30.0	1.17 (0.84–1.63)	0.346	22.9	0.269	0.059
		<30.0	0.63 (0.43–0.94)	0.023	85.6	<0.001	
	Stroke history (%)	≥30.0	0.97 (0.78–1.21)	0.805	66.5	0.004	0.233
		<30.0	0.60 (0.35–1.04)	0.067	63.9	0.040	
	CHA DS_2_- VASc score	≥4.0	0.91 (0.68–1.21)	0.506	75.9	<0.001	0.079
		<4.0	0.60 (0.40–0.90)	0.014	38.1	0.199	
	Control	VKA	0.64 (0.35–1.18)	0.153	-	-	0.523
		NOAC	0.91 (0.68–1.21)	0.506	75.9	<0.001	
		OAC	0.72 (0.43–1.21)	0.218	77.5	0.012	
	Follow-up (yrs)	≥2.0	0.77 (0.59–1.00)	0.049	76.3	<0.001	0.083
		<2.0	1.11 (0.80–1.54)	0.526	22.1	0.278	

## Data Availability

All data were obtained from the databases. The original contributions presented in this study are included in the article and Supplementary Material. Further inquiries can be directed to the corresponding author.

## Supplementary Materials

The following supporting information can be downloaded at: https://www.mdpi.com/article/10.3390/jcdd12120483/s1. Section S1: Search strategy in electronic databases; Section S2: Supplementary Tables; Table S1: Quality assessment of included RCTs; Table S2: Quality assessment of included PSM studies; Section S3: Supplementary Figures; Figure S1: Sensitivity analysis for composite endpoint; Figure S2: Meta-regression of mean age for LAA closure vs. OAC on composite endpoint (*p* = 0.359); Figure S3: Meta-regression of male proportion for LAA closure vs. OAC on composite endpoint (*p* = 0.557); Figure S4: Meta-regression of DM proportion for LAA closure vs. OAC on composite endpoint (*p* = 0.356); Figure S5: Meta-regression of hypertension proportion for LAA closure vs. OAC on composite endpoint (*p* = 0.471); Figure S6: Meta-regression of CAD proportion for LAA closure vs. OAC on composite endpoint (*p* = 0.175); Figure S7: Meta-regression of CHF proportion for LAA closure vs. OAC on composite endpoint (*p* = 0.095); Figure S8: Meta-regression of stroke proportion for LAA closure vs. OAC on composite endpoint (*p* = 0.978); Figure S9: Meta-regression of CHA DS_2_- VASc score for LAA closure vs. OAC on composite endpoint (*p* = 0.949); Figure S10: Meta-regression of class of oral anticoagulant used for LAA closure vs. OAC on composite endpoint (*p* = 0.515); Figure S11: Meta-regression of follow-up for LAA closure vs. OAC on composite endpoint (*p* = 0.913); Figure S12: Sensitivity analysis for major bleeding; Figure S13: Meta-regression of mean age for LAA closure vs. OAC on major bleeding(*p* = 0.086); Figure S14: Meta-regression of male proportion for LAA closure vs. OAC on major bleeding(*p* = 0.443); Figure S15: Meta-regression of DM proportion for LAA closure vs. OAC on major bleeding(*p* = 0.058); Figure S16: Meta-regression of hypertension proportion for LAA closure vs. OAC on major bleeding(*p* = 0.058); Figure S17: Meta-regression of CAD proportion for LAA closure vs. OAC on major bleeding(*p* = 0.437); Figure S18: Meta-regression of CHF proportion for LAA closure vs. OAC on major bleeding(*p* = 0.075); Figure S19: Meta-regression of stroke proportion for LAA closure vs. OAC on major bleeding(*p* = 0.075); Figure S20: Meta-regression of CHA DS_2_- VASc score for LAA closure vs. OAC on major bleeding (*p* = 0.454); Figure S21: Meta-regression of class of oral anticoagulant used for LAA closure vs. OAC on major bleeding (*p* = 0.812); Figure S22: Meta-regression of follow-up for LAA closure vs. OAC on major bleeding (*p* = 0.356).
