# Supplementary material for: Left Atrial Appendage Closure Versus Oral Anticoagulants in Atrial Fibrillation: A Systematic Review and Meta-Analysis
Source: J Cardiovasc Dev Dis. 2025 Dec 8;12(12):483. doi: 10.3390/jcdd12120483 (PMC12733798; doi:10.3390/jcdd12120483)
Supplement: Supplementary file 1 [file jcdd-12-00483-s001.zip › jcdd-3985526-supplementary.pdf]

Section 1. Search strategy in electronic databases:

PubMed:

("Atrial Fibrillation"[Mesh] OR "Atrial Fibrillation"[All Fields] OR AF[All Fields]) AND ("Left Atrial Appendage Closure"[Mesh] OR "Left Atrial Appendage Occlusion"[Mesh] OR "LAA Closure"[All Fields] OR "LAA Occlusion"[All Fields] OR Watchman[All Fields] OR Amplatzer[All Fields] OR LAmbre[All Fields]) AND ("Oral Anticoagulants"[Mesh] OR "Warfarin"[Mesh] OR "Vitamin K Antagonists"[Mesh] OR "NOACs"[All Fields] OR "Dabigatran"[All Fields] OR "Rivaroxaban"[All Fields] OR "Apixaban"[All Fields]) AND (("Randomized Controlled Trial"[pt] OR "RCT"[All Fields]) OR ("Propensity Score Matching"[All Fields] OR "PSM"[All Fields]))

EmBase:

("atrial fibrillation"/exp OR atrial fibrillation:ab,ti) AND ("left atrial appendage closure"/exp OR left atrial appendage closure:ab,ti OR LAA closure:ab,ti OR Watchman:ab,ti OR Amplatzer:ab,ti OR LAmbre:ab,ti) AND ("oral anticoagulant"/exp OR warfarin:ab,ti OR vitamin k antagonist:ab,ti OR NOAC:ab,ti OR dabigatran:ab,ti OR rivaroxaban:ab,ti OR apixaban:ab,ti) AND (("randomized controlled trial"/exp OR randomized controlled trial:ab,ti) OR ("propensity score matching"/exp OR propensity score matching:ab,ti))

Cochrane library:

#1 "atrial fibrillation"

#2 "left atrial appendage closure" OR "left atrial appendage occlusion" OR "LAA closure" OR "LAA occlusion" OR Watchman OR Amplatzer OR LAmbre

#3 "oral anticoagulant" OR warfarin OR vitamin k antagonist OR NOAC OR dabigatran OR rivaroxaban OR apixaban

#4 ("randomized controlled trial" OR "randomized" OR "controlled trial")

#5 ("propensity score matching" OR "PSM")

#6 (#1 AND #2 AND #3 AND (#4 OR #5))

Web of Science:

TS=("atrial fibrillation" OR AF) AND TS=("left atrial appendage closure" OR "LAA closure" OR Watchman OR Amplatzer OR LAmbre) AND TS=("oral anticoagulant*" OR warfarin OR NOAC* OR dabigatran OR rivaroxaban OR apixaban) AND (TS=("randomized controlled trial*" OR RCT) OR TS=("propensity score matching*" OR PSM))

Section 2: Supplementary Tables:

Table S1. Quality assessment of included RCTs

Study	Random sequence generation	Allocation concealment	Blinding of participants and personnel	Blinding of outcome assessment	Incomplete outcome data	Selective reporting	Other bias
Holmes 2014 [25]	Low	Low	Low	Low	Low	Low	Low
Reddy 2014 [26]	Low	Low	Low	Low	Unclear	Low	Low
Osmancik 2020 [27]	Low	Low	Low	Low	Low	Low	Low
Wazni 2025 [39]	Low	Low	Low	Low	Low	Low	Low

Table S2. Quality assessment of included PSM studies

Study	Selection				Comparability	Outcome			NOS
	Representativeness of the exposed cohort	Selection of the non exposed cohort	Ascertainment of fish consumption	Demonstration that outcomes was not present at start of study	Comparability on the basis of the design or analysis	Assessment of outcome	Adequate follow-up duration	Adequate follow-up rate	Overall score
Godino 2020 [28]	0	1	1	1	2	1	1	1	8
Nielsen-Kudsk 2021 [29]	1	1	1	1	2	1	1	1	9
Paiva 2021 [30]	0	1	1	1	2	1	1	1	8
Ding 2022 [31]	1	1	1	1	2	1	1	1	9
Noseworthy 2022 [32]	1	1	1	1	2	1	1	1	9
Caneiro-Queija 2022 [33]	0	1	1	1	2	1	1	1	8
Korsholm 2022 [34]	1	1	1	1	2	1	1	1	9
Tiosano 2023 [35]	1	1	1	1	2	1	1	1	9
Ng 2023 [36]	1	1	1	1	2	1	1	1	9
Fei 2023 [37]	1	1	1	1	2	1	1	1	9
Aglan 2024 [38]	1	1	1	1	2	1	1	1	9

Section 3. Supplementary Figures:

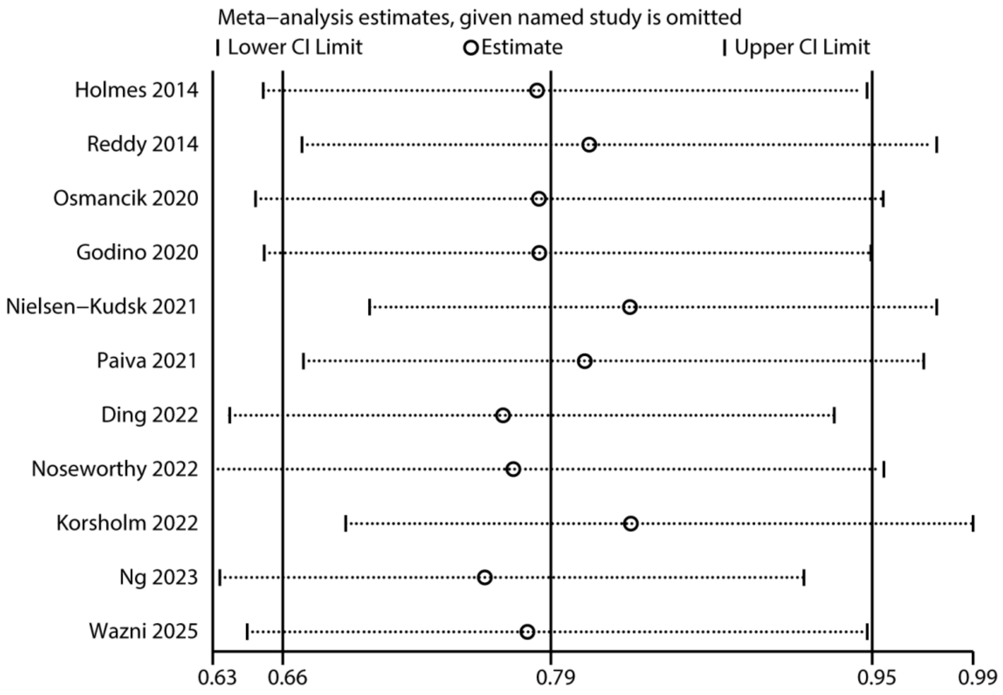

Figure S1. Sensitivity analysis for composite endpoint

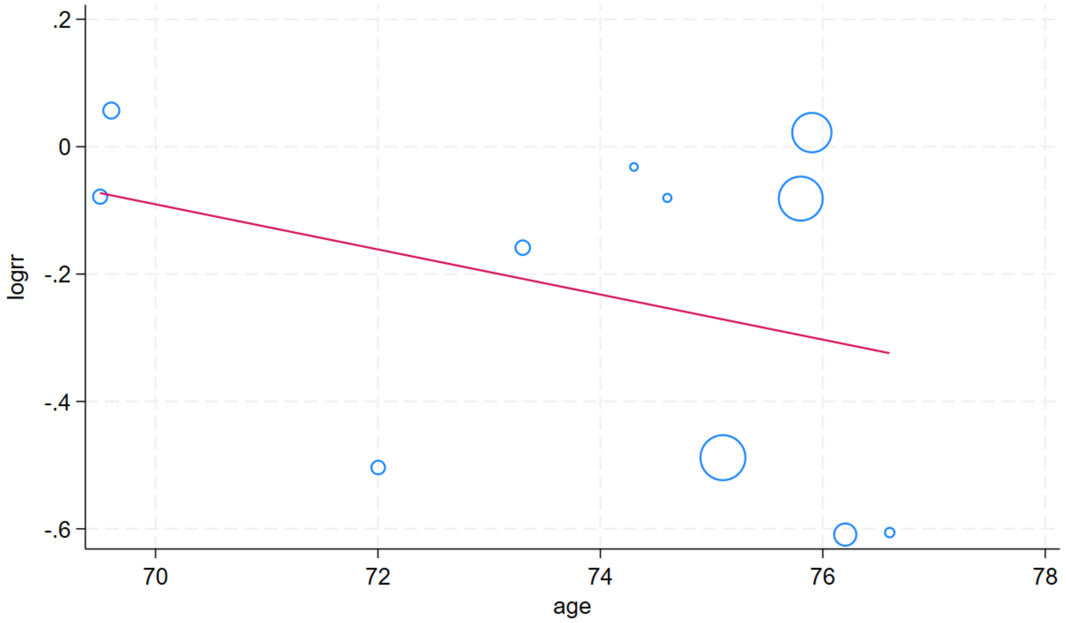

Figure S2. Meta-regression of mean age for LAA closure vs OAC on composite endpoint (P=0.359)

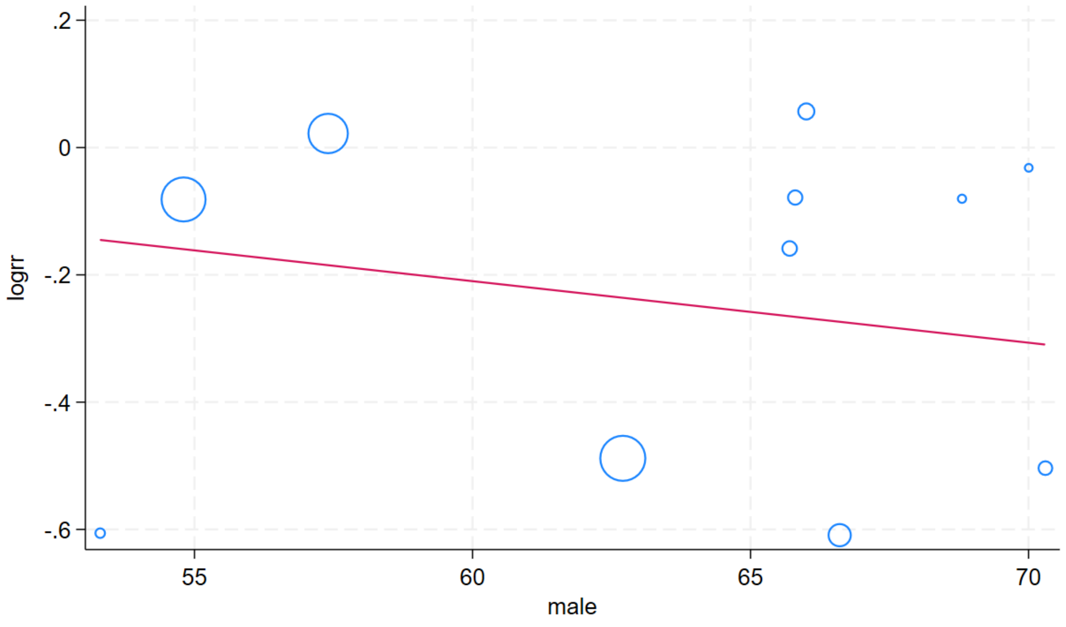

Figure S3. Meta-regression of male proportion for LAA closure vs OAC on composite endpoint (P=0.557)

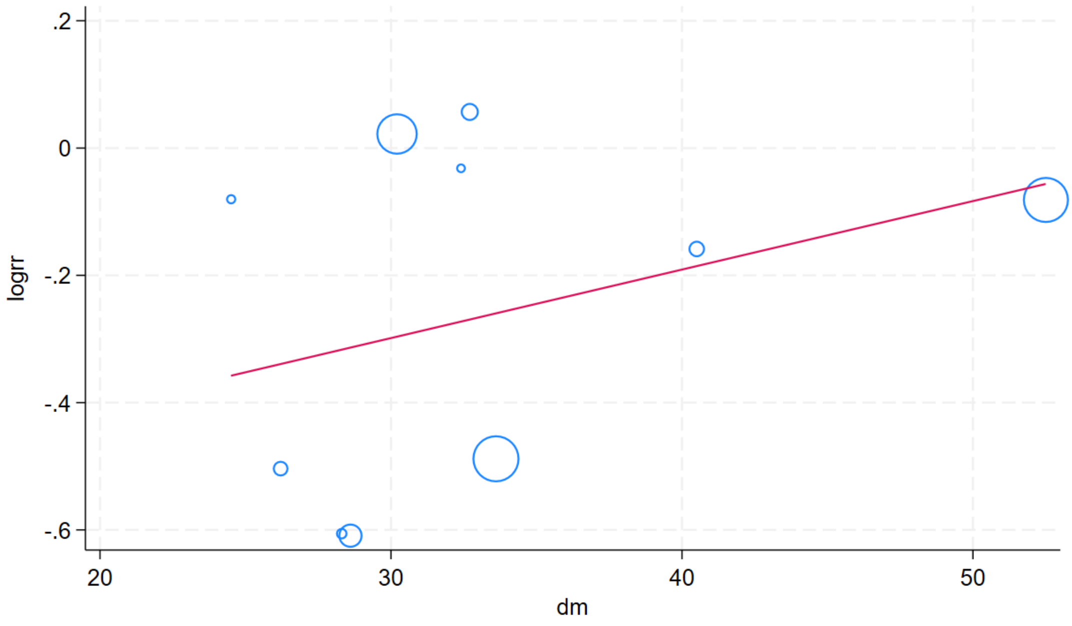

Figure S4. Meta-regression of DM proportion for LAA closure vs OAC on composite endpoint (P=0.356)

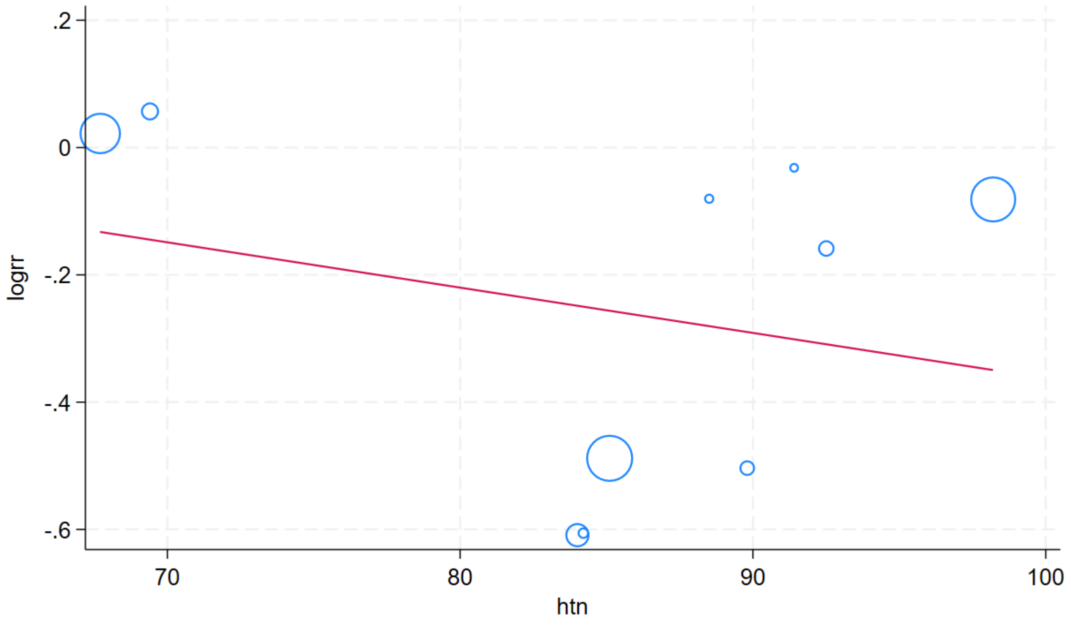

Figure S5. Meta-regression of hypertension proportion for LAA closure vs OAC on composite endpoint (P=0.471)

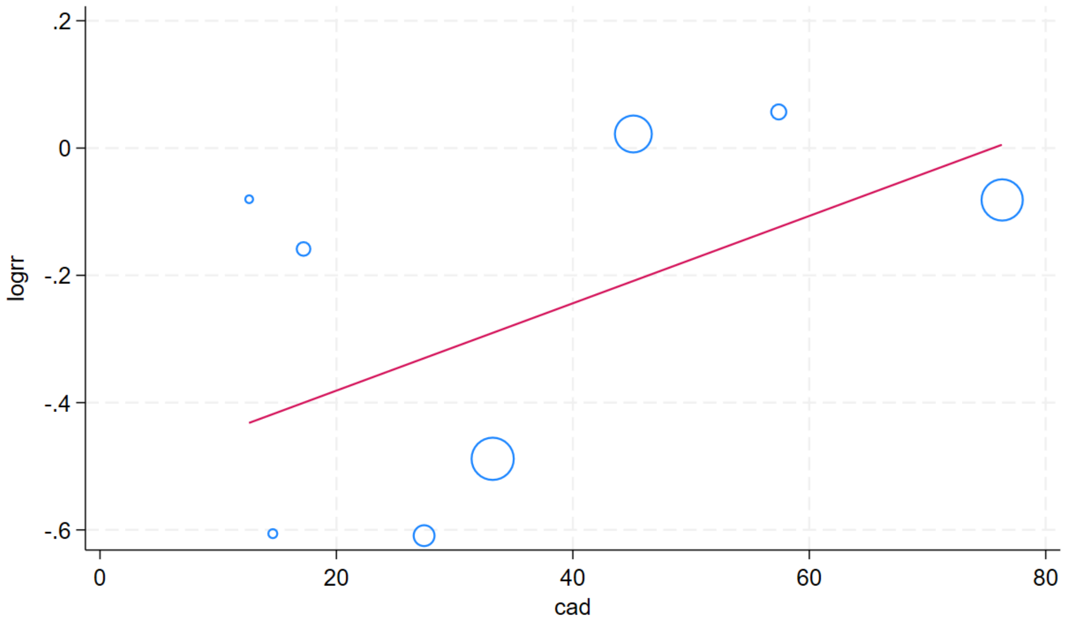

Figure S6. Meta-regression of CAD proportion for LAA closure vs OAC on composite endpoint (P=0.175)

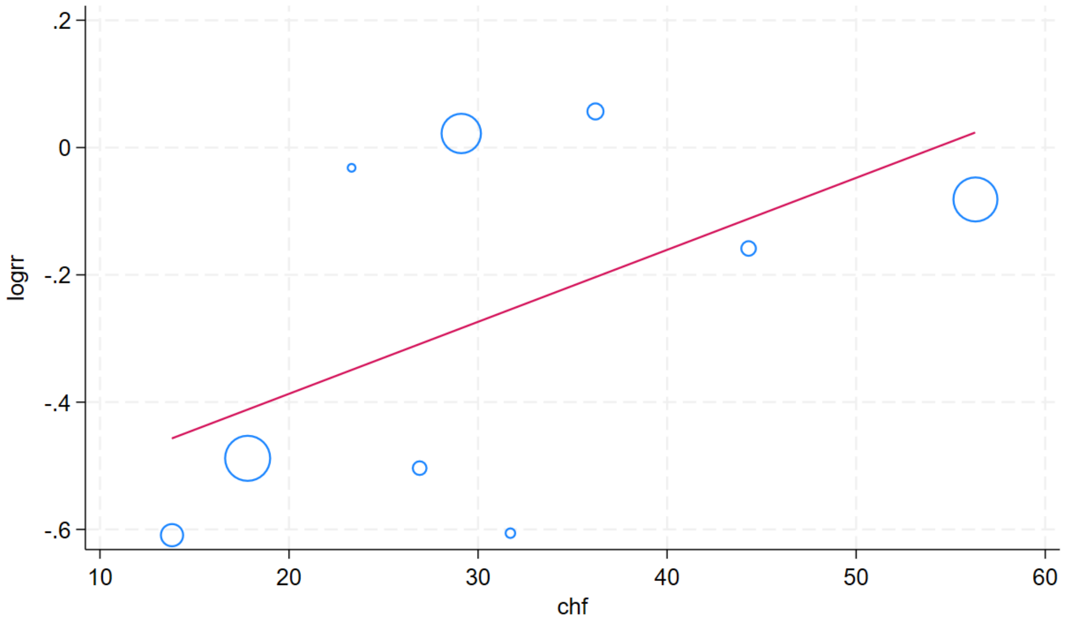

Figure S7. Meta-regression of CHF proportion for LAA closure vs OAC on composite endpoint (P=0.095)

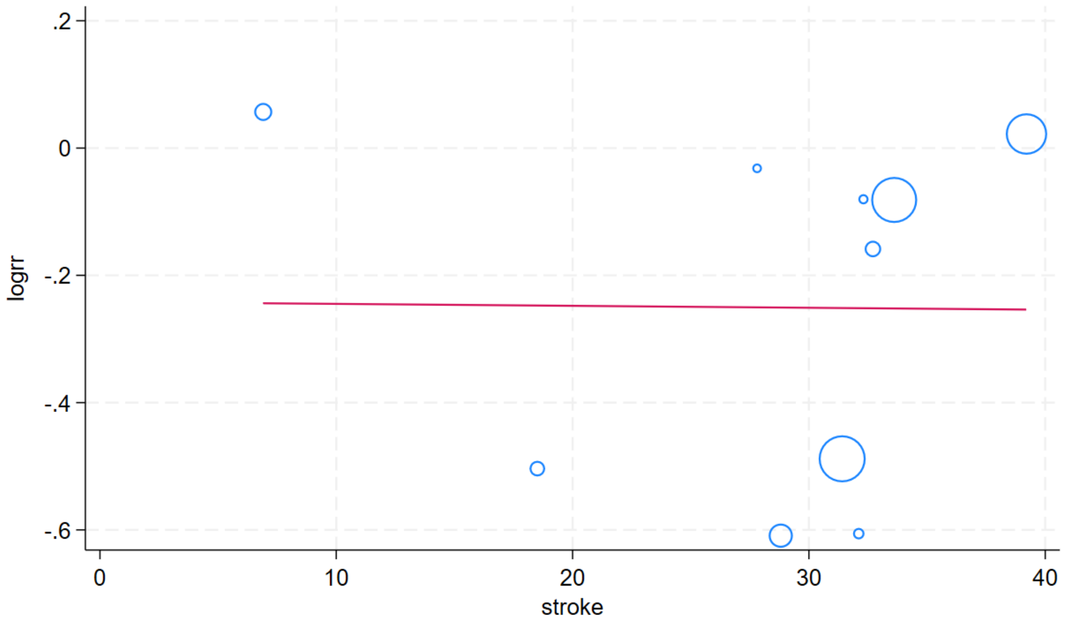

Figure S8. Meta-regression of stroke proportion for LAA closure vs OAC on composite endpoint (P=0.978)

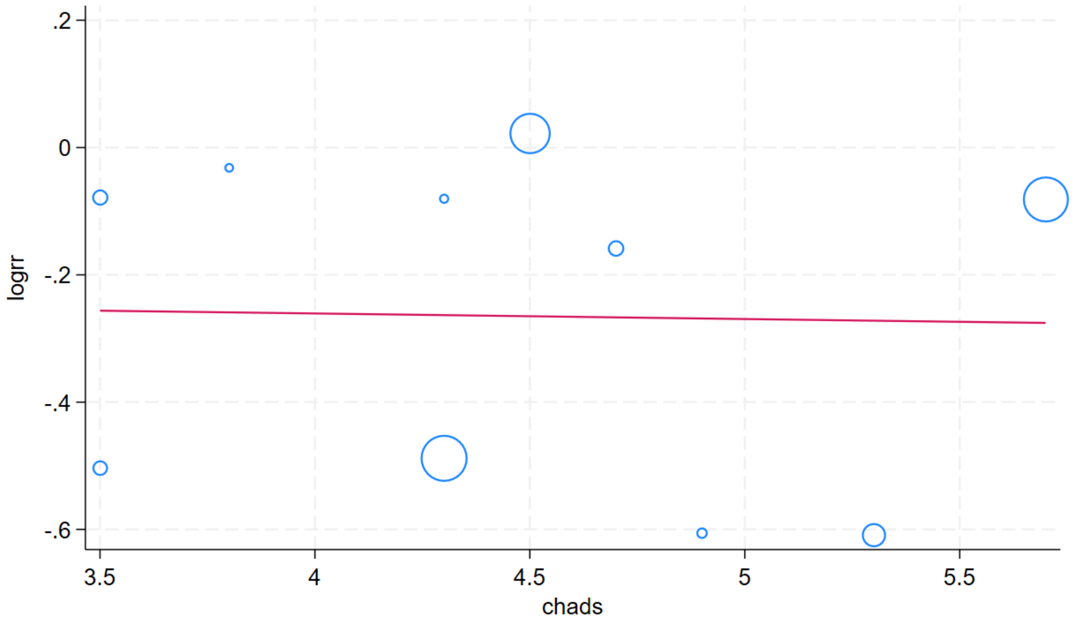

Figure S9. Meta-regression of CHA DS$_2$- VASc score for LAA closure vs OAC on composite endpoint (P=0.949)

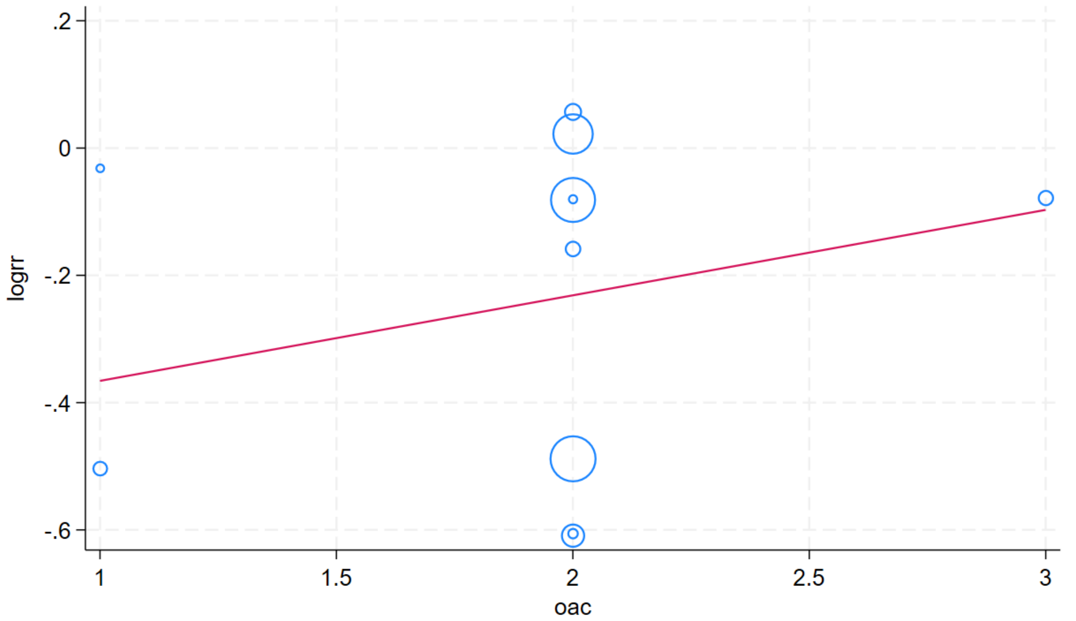

Figure S10. Meta-regression of class of oral anticoagulant used for LAA closure vs OAC on composite endpoint (P=0.515)

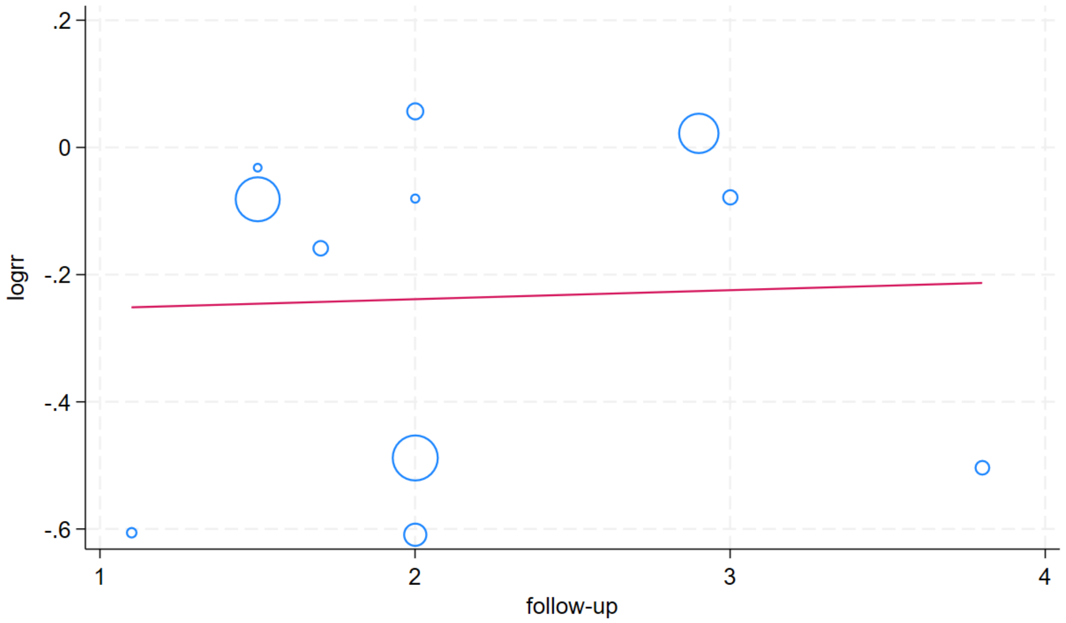

Figure S11. Meta-regression of follow-up for LAA closure vs OAC on composite endpoint (P=0.913)

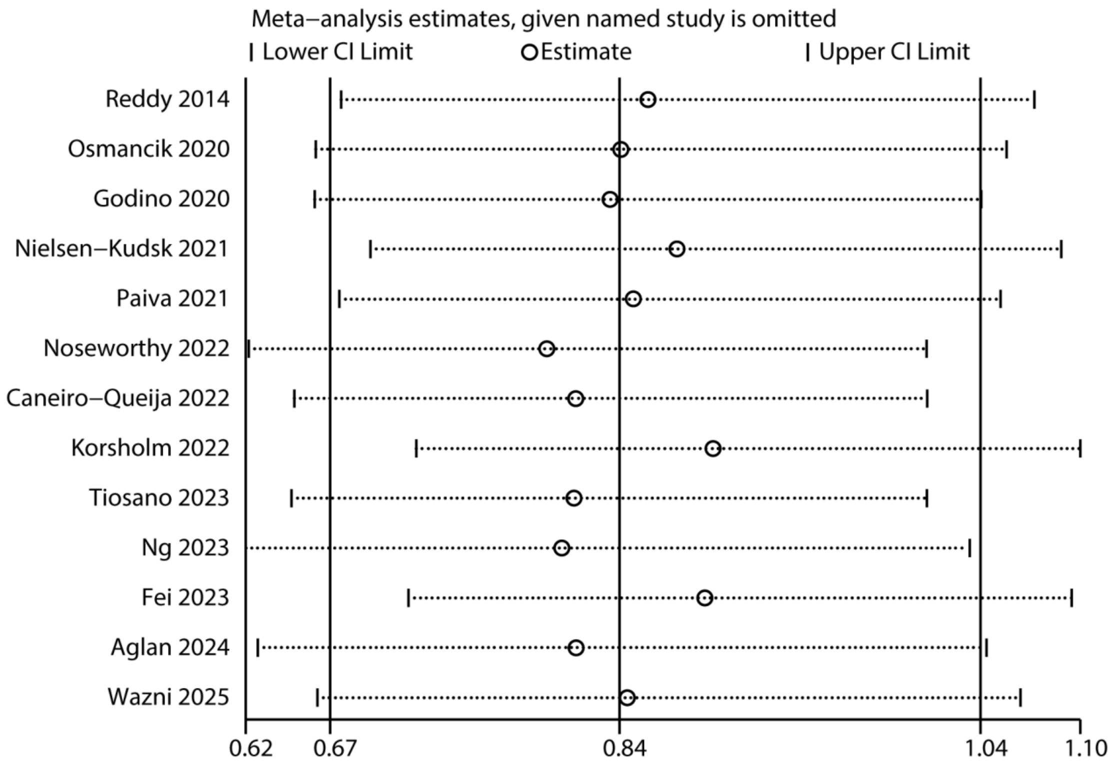

Figure S12. Sensitivity analysis for major bleeding

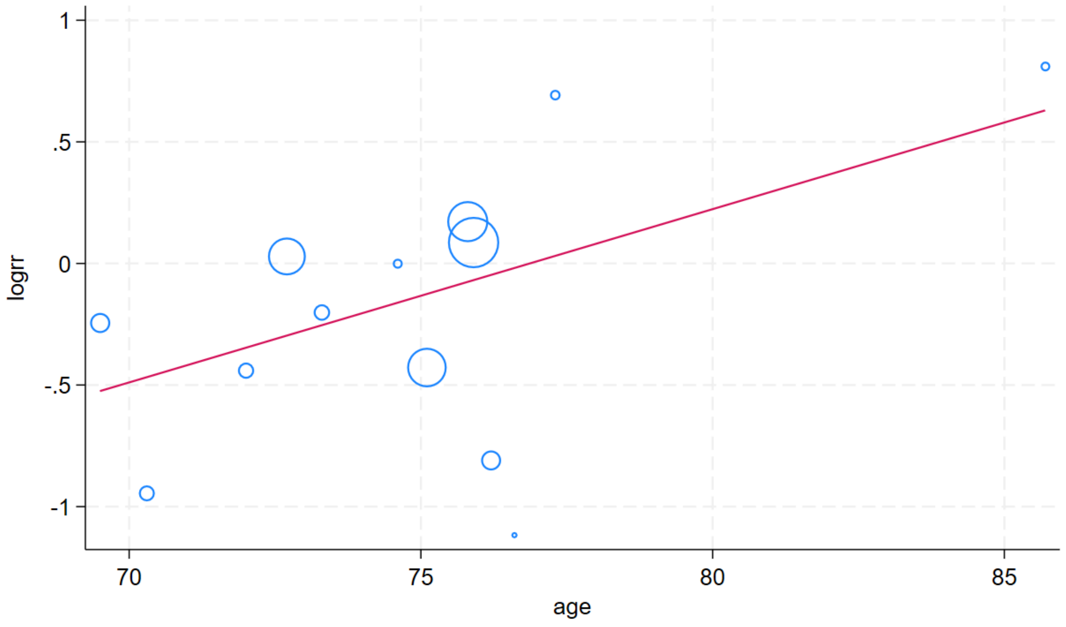

Figure S13. Meta-regression of mean age for LAA closure vs OAC on major bleeding(P=0.086)

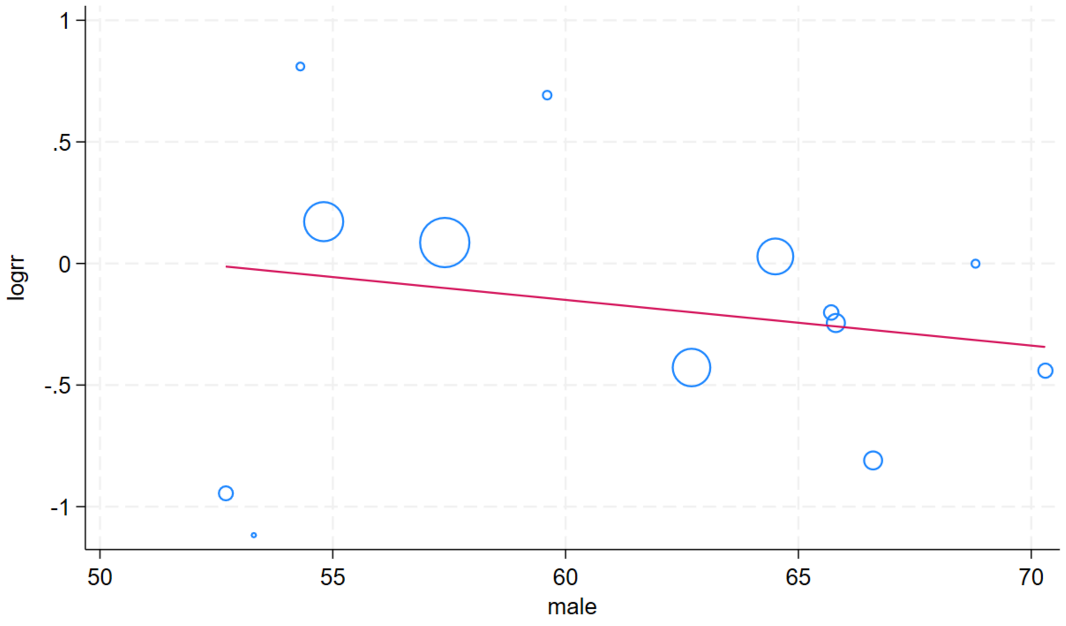

Figure S14. Meta-regression of male proportion for LAA closure vs OAC on major bleeding(P=0.443)

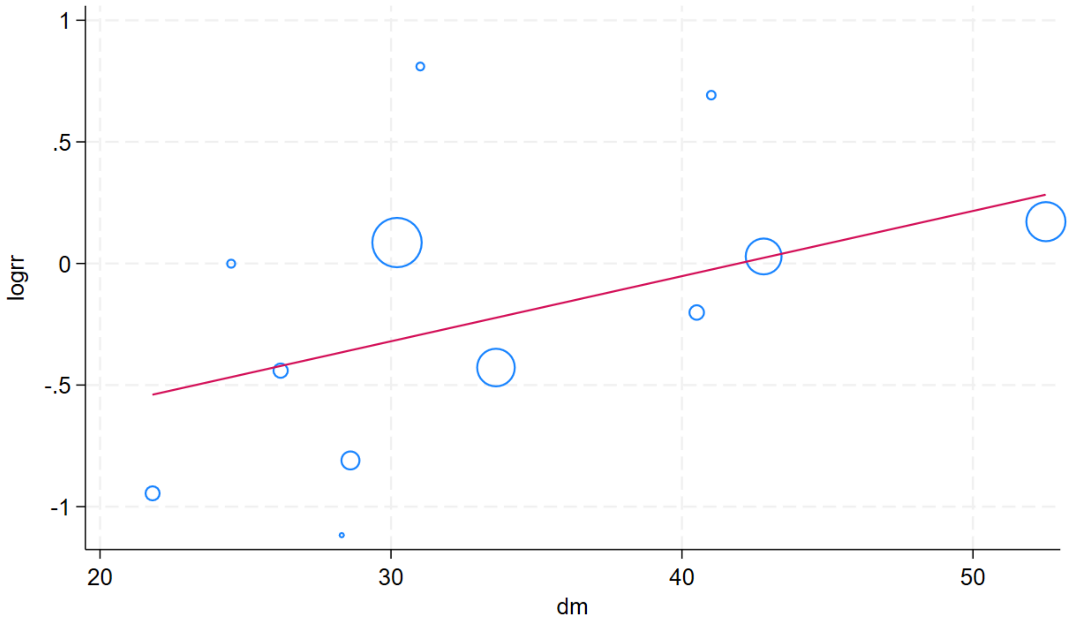

Figure S15. Meta-regression of DM proportion for LAA closure vs OAC on major bleeding(P=0.058)

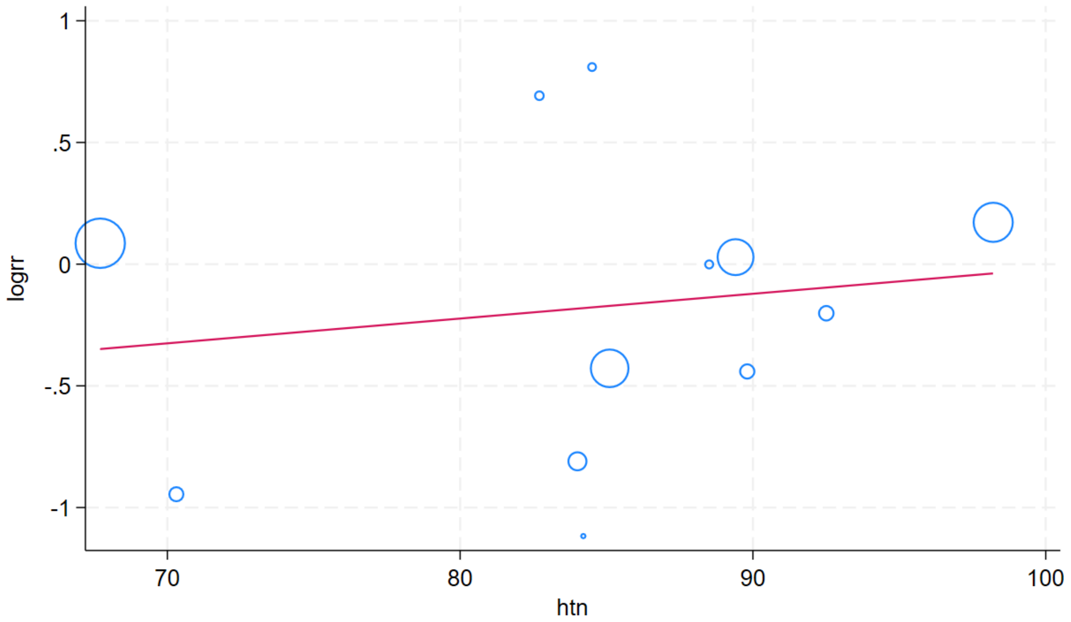

Figure S16. Meta-regression of hypertension proportion for LAA closure vs OAC on major bleeding(P=0.058)

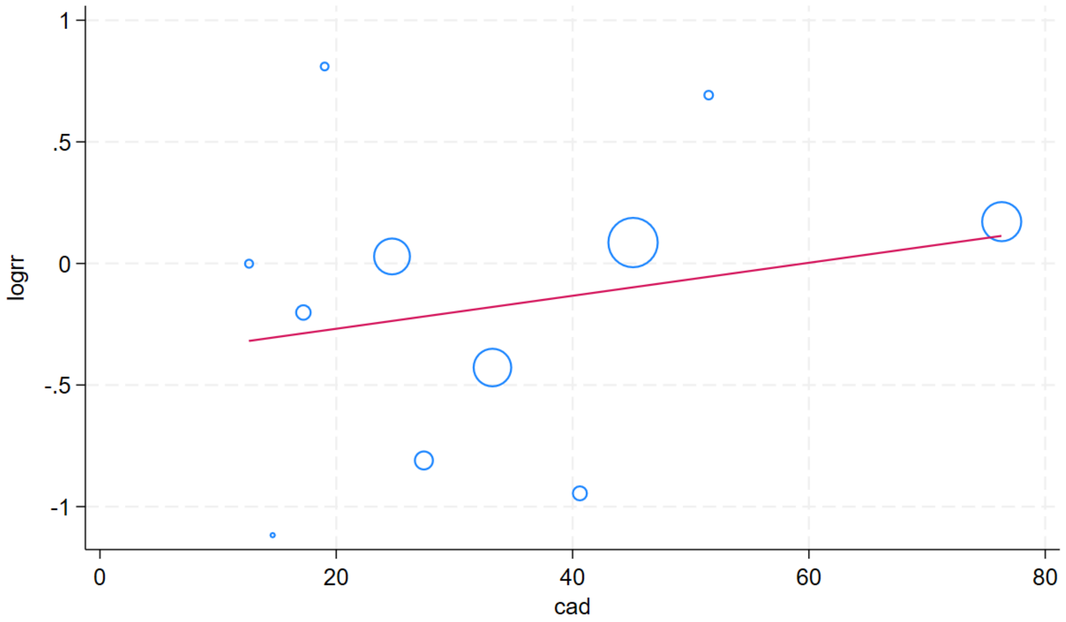

Figure S17. Meta-regression of CAD proportion for LAA closure vs OAC on major bleeding(P=0.437)

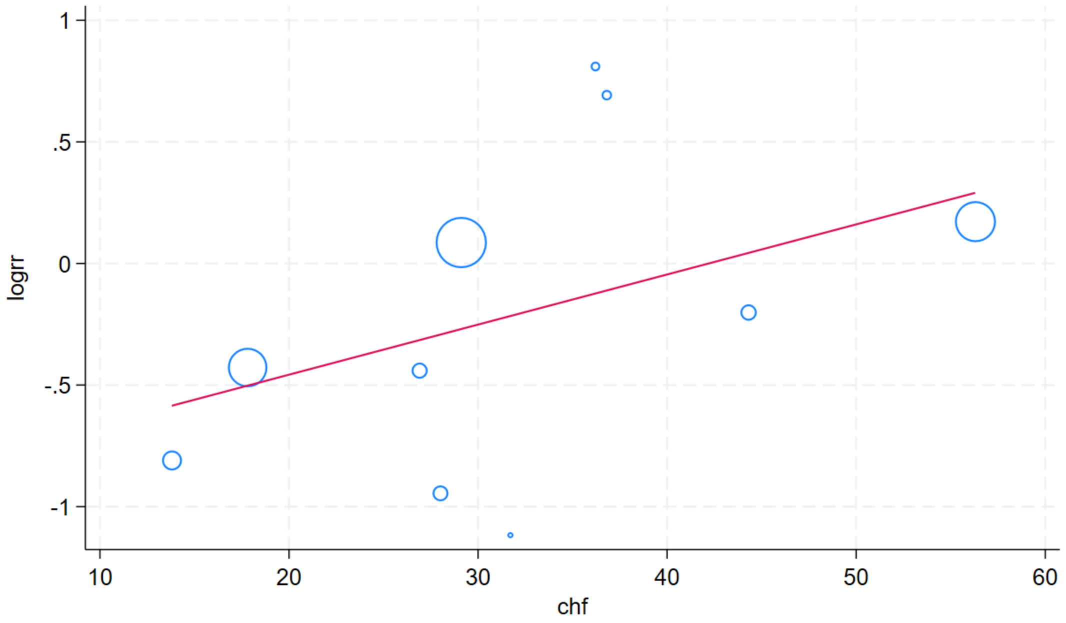

Figure S18. Meta-regression of CHF proportion for LAA closure vs OAC on major bleeding(P=0.075)

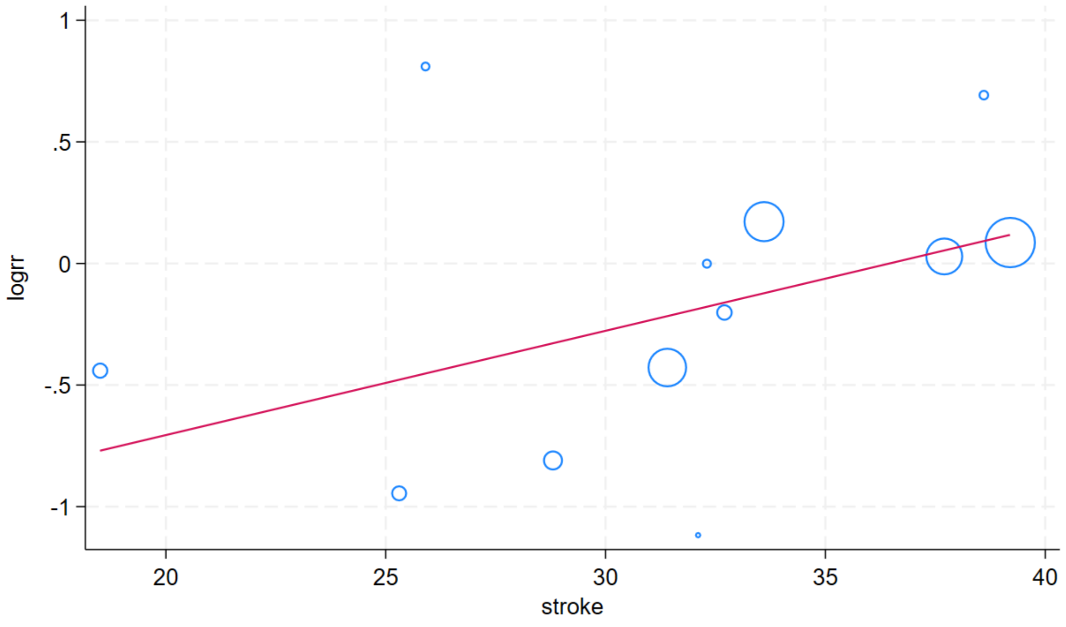

Figure S19. Meta-regression of stroke proportion for LAA closure vs OAC on major bleeding(P=0.075)

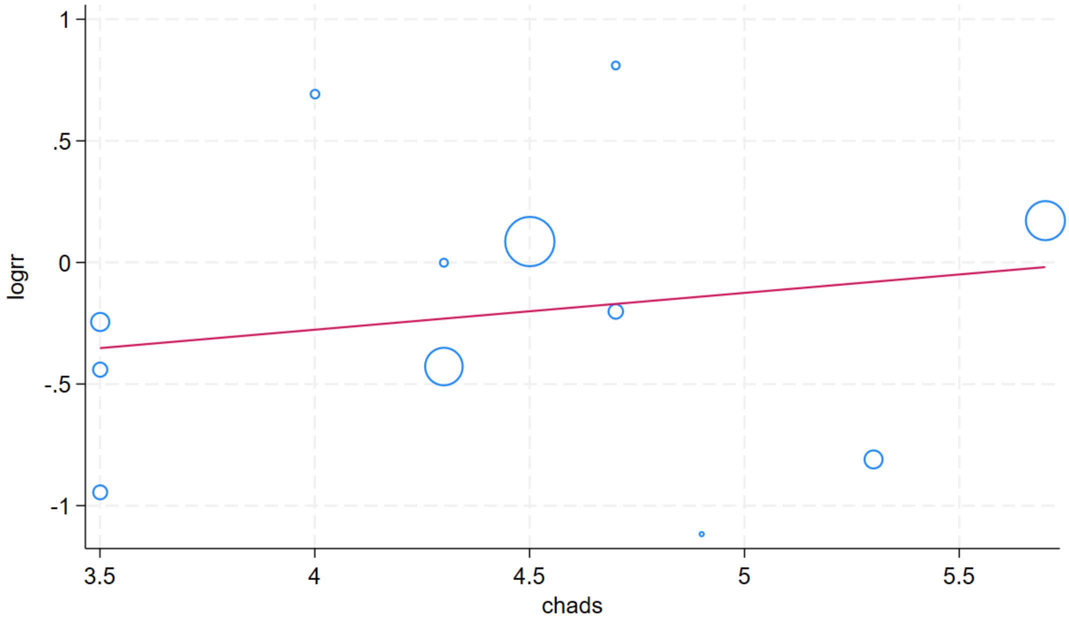

Figure S20. Meta-regression of CHA DS_2- VASc score for LAA closure vs OAC on major bleeding (P=0.454)

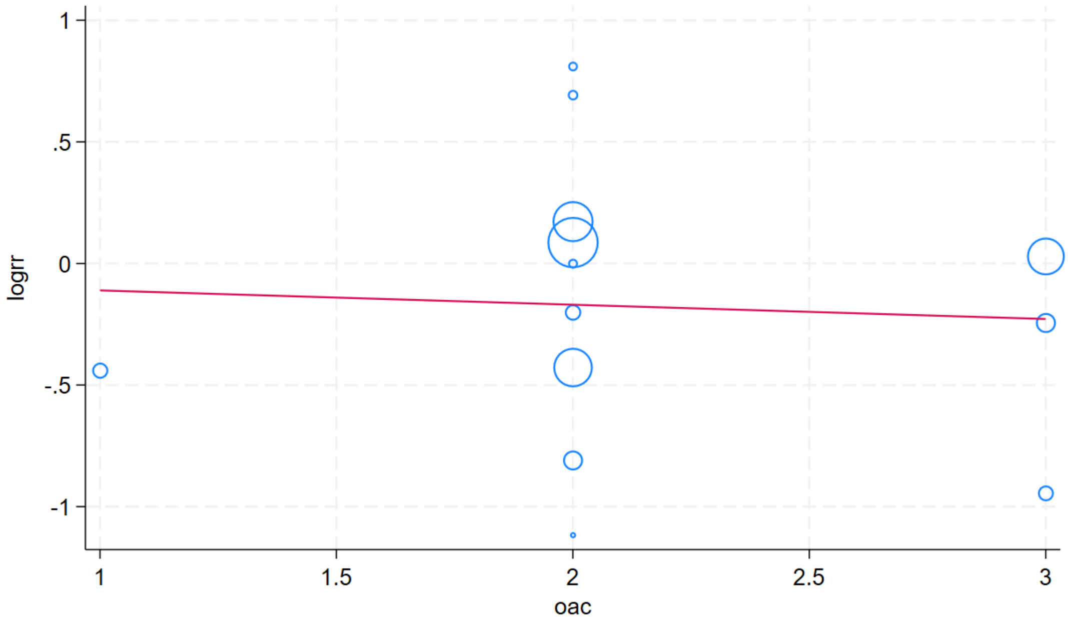

Figure S21. Meta-regression of class of oral anticoagulant used for LAA closure vs OAC on major bleeding (P=0.812)

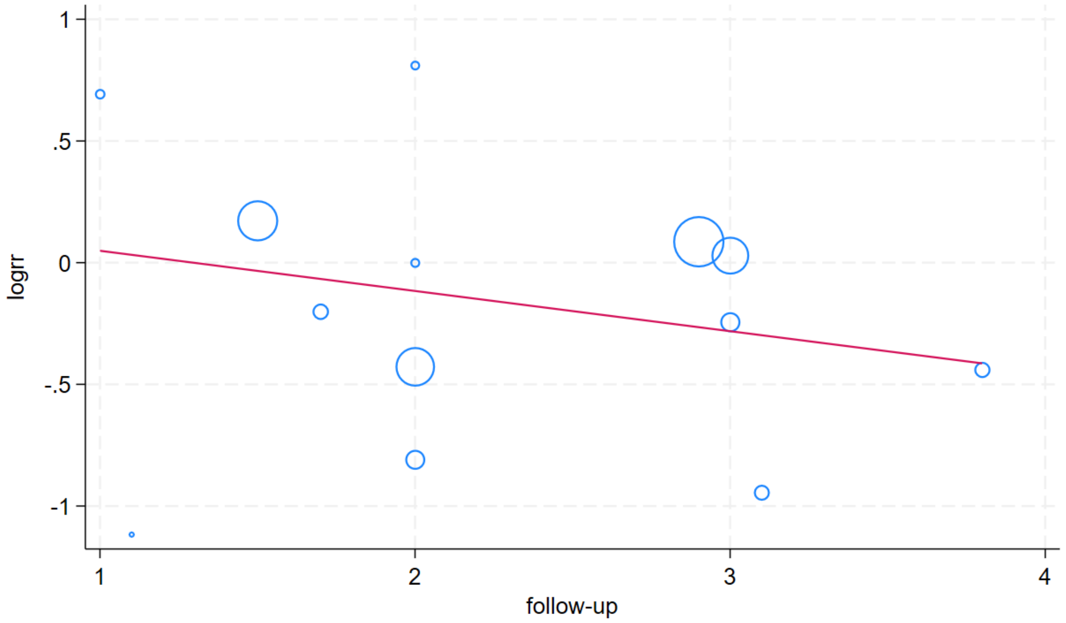

Figure S22. Meta-regression of follow-up for LAA closure vs OAC on major bleeding (P=0.356)
